# Alternative CD44 splicing identifies epithelial prostate cancer cells from the mesenchymal counterparts

**DOI:** 10.1007/s12032-015-0593-z

**Published:** 2015-04-09

**Authors:** James R. Hernandez, John J. Kim, James E. Verdone, Xin Liu, Gonzalo Torga, Kenneth J. Pienta, Steven M. Mooney

**Affiliations:** 1 Department of Urology, The James Buchanan Brady Urological Institute, Johns Hopkins University, Baltimore, MD 21287 USA; 2 Department of Oncology, Johns Hopkins University, Baltimore, MD USA; 3 Department of Pharmacology and Molecular Sciences, Johns Hopkins University, Baltimore, MD USA; 4 Department of Chemical and Biomolecular Engineering, Johns Hopkins University, Baltimore, MD USA; 5 Department of Biological Chemistry, Johns Hopkins University, Baltimore, MD USA; 6 Department of Bioengineering, University of California (UC), Berkeley, USA; 7 The UC Berkeley–UC San Francisco Graduate Program in Bioengineering, UC Berkeley, Berkeley, CA USA

**Keywords:** Epithelial to mesenchymal transition (EMT), CD44, OVOL1/2, ZEB1, E-cadherin (CDH1), RBM35A/ESRP1 epithelial splicing regulatory protein 1

## Abstract

An epithelial to mesenchymal transition (EMT) has been shown to be a necessary precursor to prostate cancer metastasis. Additionally, the differential expression and splicing of mRNAs has been identified as a key means to distinguish epithelial from mesenchymal cells by qPCR, western blotting and immunohistochemistry. However, few markers exist to differentiate between these cells by flow cytometry. We previously developed two cell lines, PC3-Epi (epithelial) and PC3-EMT (mesenchymal). RNAseq was used to determine the differential expression of membrane proteins on PC3-Epi/EMT. We used western blotting, qPCR and flow cytometry to validate the RNAseq results. CD44 was one of six membrane proteins found to be differentially spliced between epithelial and mesenchymal PC3 cells. Although total CD44 was positive in all PC3-Epi/EMT cells, PC3-Epi cells had a higher level of CD44v6 (CD44 variant exon 6). CD44v6 was able to differentiate epithelial from mesenchymal prostate cancer cells using either flow cytometry, western blotting or qPCR.

**Electronic supplementary material:**

The online version of this article (doi:10.1007/s12032-015-0593-z) contains supplementary material, which is available to authorized users.

## Introduction

Epithelial to mesenchymal transition has been shown to be a necessary step in the process of forming metastasis [1, 2]. In order to characterize these cells in the background of a single genome, an epithelial clone of PC3 cells, PC3-Epi, was isolated along with a mesenchymal derivative of PC3 known as PC3-EMT. Although PC3-Epi and PC3-EMT have similar growth rates when grown in subcutaneous mouse xenografts, PC3-EMT had a fourfold higher rate in their ability to form multiple metastatic lesions after mouse intracardial injection. Additionally, both cell lines undergo EMT and the reverse process of MET within these sites of metastasis as evidenced by ZEB1 (mesenchymal) and E-cadherin (epithelial) immunohistochemical staining [3]. In order to isolate epithelial and mesenchymal prostate cells within the patient bone marrow without fixing them, flow cytometric methods need to be developed. As stated, ZEB1 and E-cadherin are excellent markers; however, ZEB1 requires fixation of cells and E-cadherin is not specific enough to use as a stand-alone marker for metastatic prostate cancer. Here, we will demonstrate that RNA-seq of the PC3-Epi and PC3-EMT cell lines suggests that CD44 would be a good candidate.

The hyaluronic acid receptor CD44 and its many isoforms are associated with a wide variety of cell types including epithelial, mesenchymal and CSCs (cancer stem cells). Previous studies have also shown that these isoforms are related to various survival functions and tumorigenesis [4]. Therefore, in order to differentiate between cell types, it is critical to understand and distinguish among the numerous isoforms of CD44. The CD44 gene consists of 20 exons and is subject to significant alternative splicing [5]. Within the extracellular domain of CD44, there exists a highly variable 10 exon region and it is the splicing pattern of these 10 exons that defines a given isoform. Typically, CD44 is categorized into one of two groups: those expressing different combinations of these 10 variant exons (CD44v) and those that do not (CD44s). Each group has been demonstrated to be linked to different functions, pathways and cell types. CD44s is typically associated with mesenchymal stem cells, while the various CD44v forms are related to hematopoietic stem cells and increased cell adhesion. However, both CD44s and CD44v have been linked to the CSC niche and cancer progression [6, 7]. The epithelial to mesenchymal transition (EMT) process whereby cancer cells lose polarity, cell–cell adhesion and gain an invasive capacity is essential for metastasis in a wide range of cancers [8]. CD44 splicing from variant (CD44v) to the standard (CD44s) form has been shown to be essential for an EMT in breast cancer [9, 10]. This up-regulation in the CD44s isoform is caused by decreased expression of epithelial splicing regulatory protein 1 (ESRP1). ESRP1 is the splicing factor for variant forms of CD44; it splices CD44 by binding to intronic regions. By itself, expression of ESRP1 prevents EMT [11]. Despite the knowledge of the various isoforms and their unique functions, total CD44 expression is most often used as a surface marker for cell stemness rather than assaying for any specific variant isoform [12].

Previously, our laboratory demonstrated that epithelial cancer cells undergo an EMT upon exposure to M2 macrophages [3, 13]. In order to obtain a purely epithelial PCa population, our laboratory isolated a single cell clone of PC3 that had high E-cadherin and low vimentin expression, denoted PC3-Epi. Alternatively, PC3 cells were incubated with M2 macrophages, which caused a stable EMT to occur after only a few days in culture and were denoted PC3-EMT. The EMT process was found to necessitate the down-regulation of the OVOL1 and OVOL2 transcription factors and up-regulation of ZEB1. Additionally, ESRP1 was most highly positively correlated with the expression of OVOL1/2 (*r* = 0.76, 0.84 respectively) in a series of 917 cancer cell lines. All this indicates that CD44 splicing maybe a tractable cell surface marker for differentiating epithelial from mesenchymal cells.

## Materials and methods

### Cancer cell line encyclopedia

Data from the Barretina group of 917 cell lines were analyzed with oncomine.org for coexpression. Previously, these cell lines from various cancers were sequenced at the DNA and RNA levels. RNA expression levels were measured using Affymetrix GeneChip Human Genome U133 Plus 2.0 Arrays [14].

### RNA isolation

RNA was isolated from cells at ~80 % confluency using an RNeasy kit (Qiagen) and treated with DNase to remove genomic DNA (Qiagen). RNA quality and concentration was determined by NanoDrop 2000 [15].

### Microarray

Microarray analysis of PC3-Epi and PC3-EMT was conducted on isolated mRNA using standard protocols by the University of Michigan Microarray Core for the GeneChip Human U133 Plus 2.0 (Affymetrix). Analysis used the bioconductor’s “Limma” package. The data file “Expression profile from PC3-Epi and derived cell lines” is accessible with the GEO ID: GSE43489 [3].

### qPCR

cDNA was prepared using the iSCRIPT kit (Bio-Rad). Quantitative analysis was performed using TaqMan Gene Expression Assays (Applied Biosystems) with an ABI 7900 HT. The following primers/probes were used: β-actin 4352935E,OVOL1 Hs00190060_m1, OVOL2 Hs00221902_m1, ZEB1 Hs00232783_m1, E-cad (CDH1), Hs01023894_m1, TWIST1 Hs01675818_s1, ZEB2 Hs00207691_m1, Snail (SNAI1) Hs00195591_m1, Slug (SNAI2) Hs00950344_m1 and ESRP1 Hs00214472_m1. Relative expression calculations were done with at least three biological replicates according to the procedures in our previous publication [3].

### Western blot

Protein extracts were prepared using Frackleton-lysis buffer [16] with protease inhibitors (Thermo Scientific 78410), and samples were electrophoresed on 4–15 % SDS-PAGE (Bio-Rad), transferred to a nitrocellulose membrane and blocked with casein blocking buffer (Sigma B6429). The list of antibodies used for western blotting is in Supplemental Table 1. The Licor Odyssey fluorescence scanner was used for visualizing the westerns.

### Flow cytometry

Flow cytometry analysis of PC3-Epi and PC3-EMT was conducted as previously described with an S3 cell sorter (Bio-Rad) using antibodies listed in Supplemental Table 2 [3, 17].

## Results

### PC3-Epi and PC3-EMT can be discriminated by western, FACs and qPCR

**Fig. 1:**
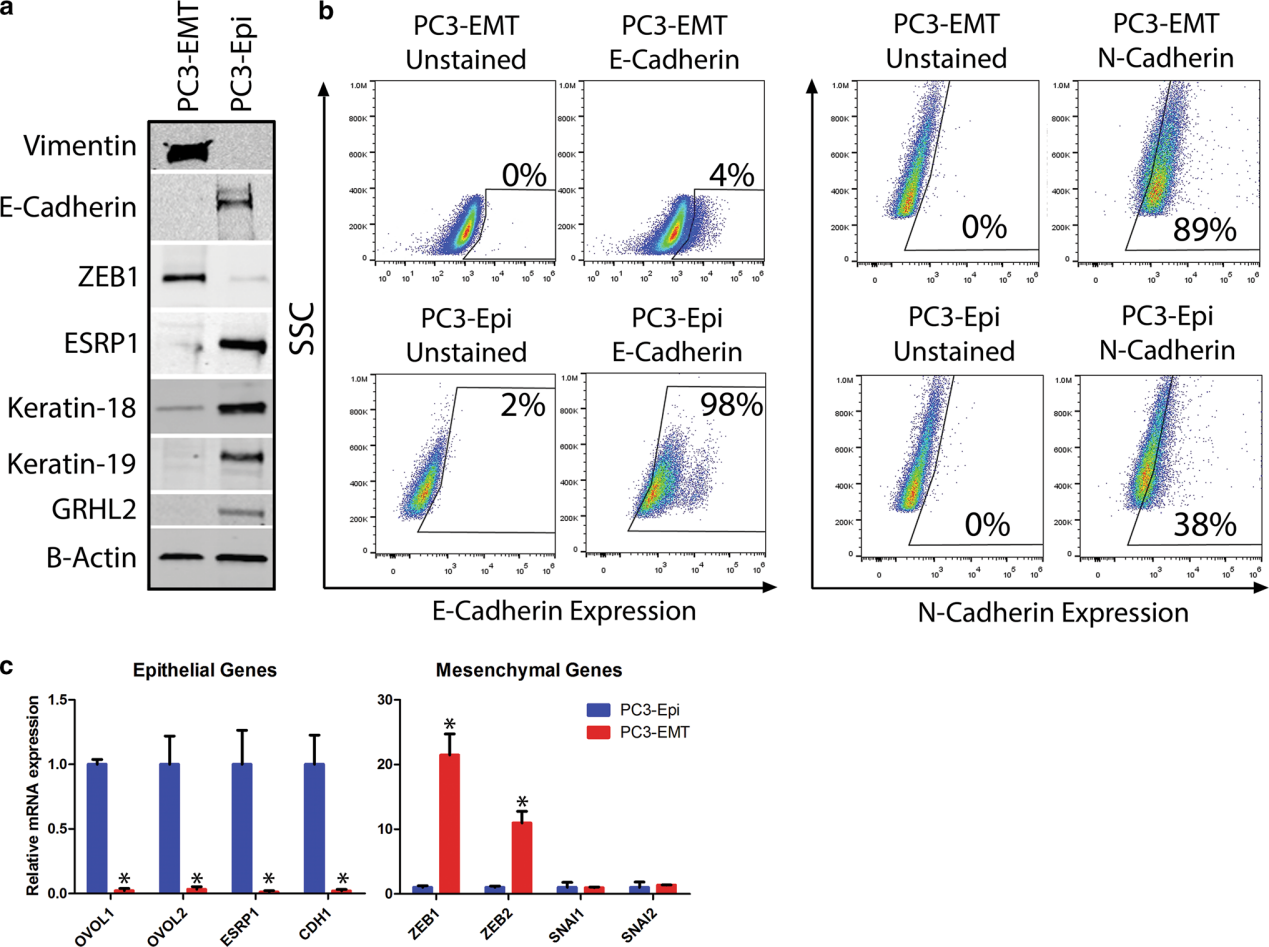
PC3-Epi and PC3-EMT are models of epithelial/mesenchymal cancer. **a** Immunoblot depicting protein expression differences in epithelial and mesenchymal genes in PC3-Epi and PC3-EMT cell lines. **b** FACS analysis of cell surface proteins E-cadherin (epithelial marker) and N-cadherin (mesenchymal marker). Data are shown with unstained controls, which are used to determine autofluorescence. **c** qPCR results depicting mRNA expression levels of epithelial and mesenchymal associated genes in PC3-Epi and PC3-EMT cell lines. Results were normalized to β-actin; *p* values are represented as *asterisk* *p* < 0.05

The PC3 parental cell line consists of a mixture of cell clones containing vimentin (mesenchymal) and E-cadherin (epithelial) positive cells (Supplemental Fig. 1). Coculturing the parental cell line with M2-macrophages resulted in induction of a stable mesenchymal cell line, PC3-EMT, exhibiting high expression of ZEB1 and vimentin (Fig. 1a) [3]. The PC3-Epi clonal population was subsequently isolated as a stable cell clone of PC3 exhibiting high expression of various epithelial proteins including E-cadherin, ESRP1, GRHL2, keratin-18 and keratin-19. PC3-Epi also was flow sortable with respect to E-cadherin: however, unexpectedly, PC3-Epi had only about 40 % positivity for cell surface E-cadherin expression, making it a poor marker for the epithelial population overall (Fig. 1b). The qPCR analysis revealed that the epithelial-specific transcription factors, OVOL1/2, were expressed over 100-fold more strongly in PC3-Epi, while PC3-EMT had high expression of ZEB1/2 (Fig. 1c). Interestingly, the CD44s splicing factor, RBM3 [18], was unchanged, while the CD44 variant splicing factor ESRP1 was dramatically up-regulated in PC-3-Epi cells (Fig. 1c).

### CSC markers CD44 and ALDH are expressed in all PC3-Epi/EMT

**Fig. 2:**
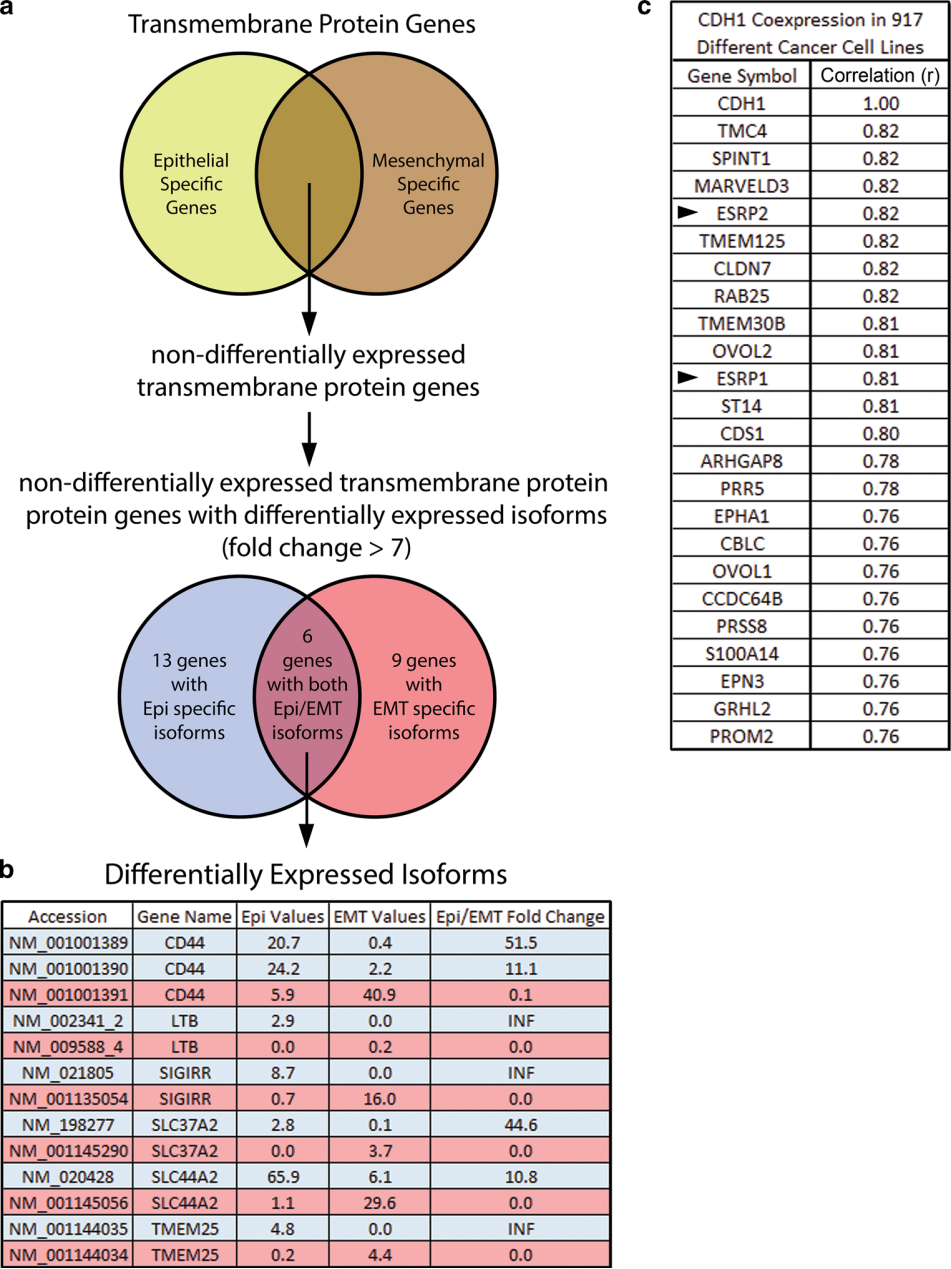
mRNA expression of epithelial and mesenchymal markers. **a** Venn diagram representing mRNA sequencing data depicting transmembrane protein genes that were not differentially expressed, but showed a sevenfold or more differential expression strictly on the isoform level. Thirteen genes were found to have epithelial-specific isoforms without having complementary isoforms in PC3-EMT. Nine genes were found to have mesenchymal-specific isoforms without having differentially expressed epithelial isoforms. Six transmembrane protein genes expressed both an epithelial and mesenchymal isoforms. **b** Table illustrating the differentially expressed isoforms found in the six genes found in the intersection of the Venn diagram. Expression is shown as fold change (PC3-Epi/PC3-EMT), with INF representing PC3-EMT expression being 0. All data shown have *p* < 0.05. **c** E-cadherin (CDH1) microarray coexpression analysis from Oncomine across 917 different cancer cell lines. Genes with a Pearson correlation greater than 0.76 are shown

Further analysis of the PC3-Epi and PC3-EMT cell lines by RNA sequencing revealed six surface proteins that were not differentially expressed at the total mRNA level, but did show differential expression of their isoforms (Fig. 2a, b). These six genes could serve as new potential surface marker proteins that could be used to differentiate between the various epithelial and mesenchymal cell types. One of the genes identified was CD44. Indeed, RNA sequencing revealed that 3 isoforms, CD44v8-10, CD44v3-10 and CD44v2-10 are expressed more highly in PC3-Epi, while the standard form, CD44s, is more highly expressed in PC3-EMT (Fig. 2b). Analysis of genes that are coexpressed with CDH1 (E-cadherin) in microarrays across 917 different cell lines from the cancer cell line encyclopedia demonstrates that the CD44 splicing protein, ESRP1, is one of the most highly correlated genes with CDH1 expression (Fig. 2c). These data, in conjunction with the high expression levels of ESRP1 in PC3-Epi and lack of expression in PC3-EMT, suggested that the alternative splice forms of CD44 may be of interest in differentiating between epithelial and mesenchymal cancer cells.

**Fig. 3:**
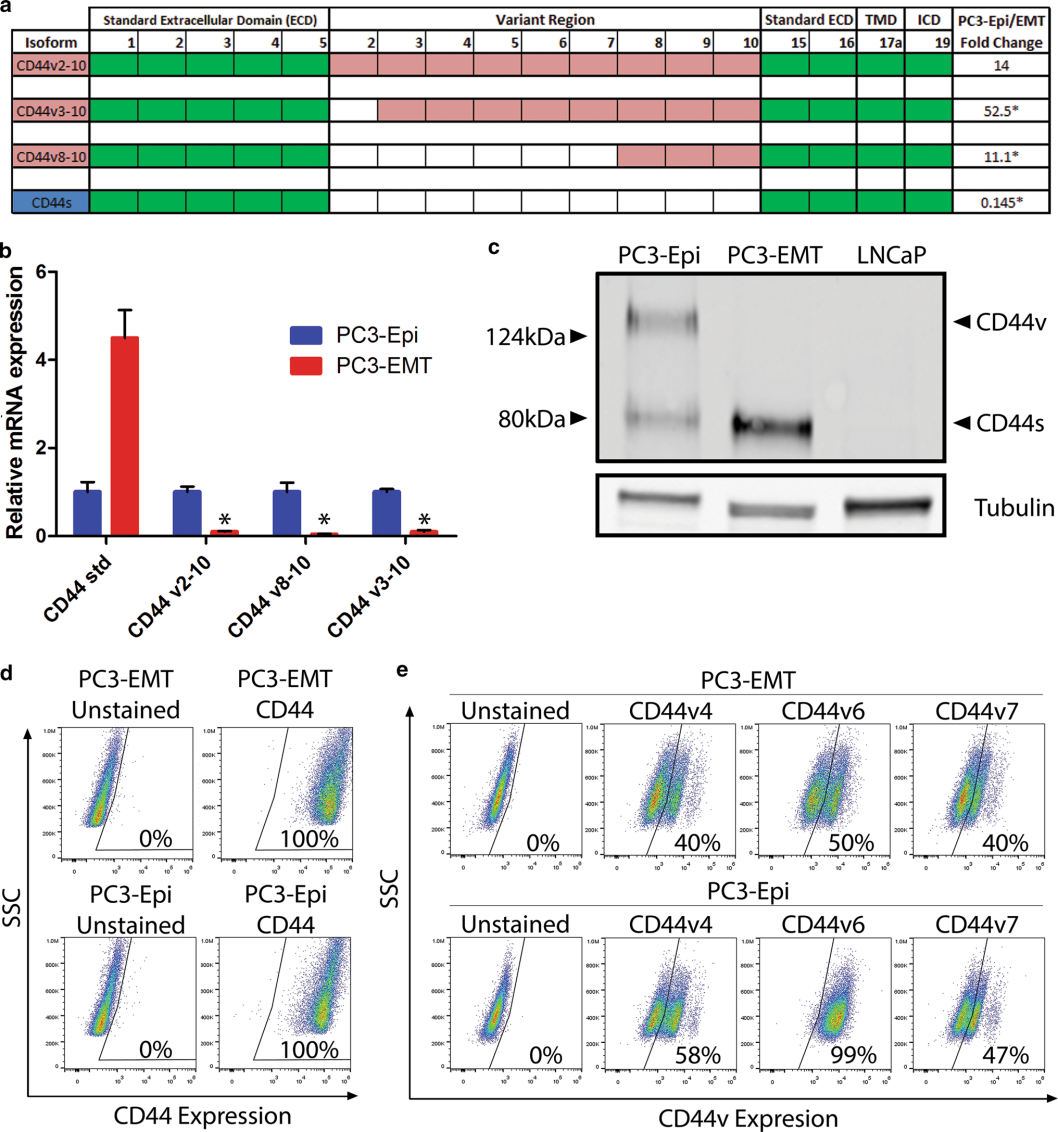
CD44 isoform expression in PC3-Epi and PC3-EMT. **a** RNA sequencing results depicting the differentially expressed CD44 isoforms in PC3-Epi and PC3-EMT. Extracellular domain is comprised of 16 exons, seven standard (*green*) and nine variant exons (*red*). The remaining the transmembrane (TMD) and intracellular domains (ICD) are standard exons expressed in all isoforms (shown also in *green*). Differential expression is shown as fold difference between PC3-Epi and PC3-EMT (represented by PC3-Epi/PC3-EMT) with *asterisk* *p* < 0.05. **b** qPCR analysis of CD44 isoform expression in PC3-Epi and PC3-EMT. Results were normalized to β-actin; *p* values are represented as *asterisk* *p* < 0.05. **c** Immunoblot for CD44 general expression in PC3-Epi and PC3-EMT. The lower molecular weight standard and higher molecular weight variant isoforms are annotated **d**, **e** FACS analysis of total CD44 or CD44 variant (CD44v4, 6, or 7) cell surface expression in PC3-Epi and PC3-EMT. Unstained cell controls were used to determine cellular autoflorescence

Analysis by RNAseq and qPCR confirm that CD44 is differentially expressed in PC3-Epi/EMT (Fig. 3a, b). Immunoblotting revealed the presence of two forms of CD44 in PC3-Epi, but only a single band in PC3-EMT (Fig. 3c). Based on the differing molecular weights, the western blot confirms the differential expression between the PC3-Epi and PC3-EMT cell lines. Analysis by flow cytometry revealed that both PC3-EMT and PC3-Epi are 100 % positive for total CD44 expression (Fig. 3d). In contrast to PC3, LNCaP cells were determined to be CD44 negative via qPCR and general CD44 expression shown in western blot and FACS analysis (Supplemental Fig. 2a-b). When investigating the CD44 isoforms containing variant exons 4, 6 or 7 with FACS, there was a much less dramatic difference in expression between PC3-Epi and EMT relative to the RNA expression data (Fig. 3e). Interestingly, a large percentage of PC3-EMT were positive for these variant exons and, relative to PC3-EMT, PC3-Epi displayed only slightly higher expression levels of variant 4 and 7. However, variant exon 6 was highly differentially expressed with virtually all PC3-Epi cells positive and 50 % of PC3-EMT positive. Again, LNCaP was used as a negative control to test specificity of the exon-specific antibodies (Supplemental Fig. 2c).

### Cell plasticity and CD44v expression

**Fig. 4:**
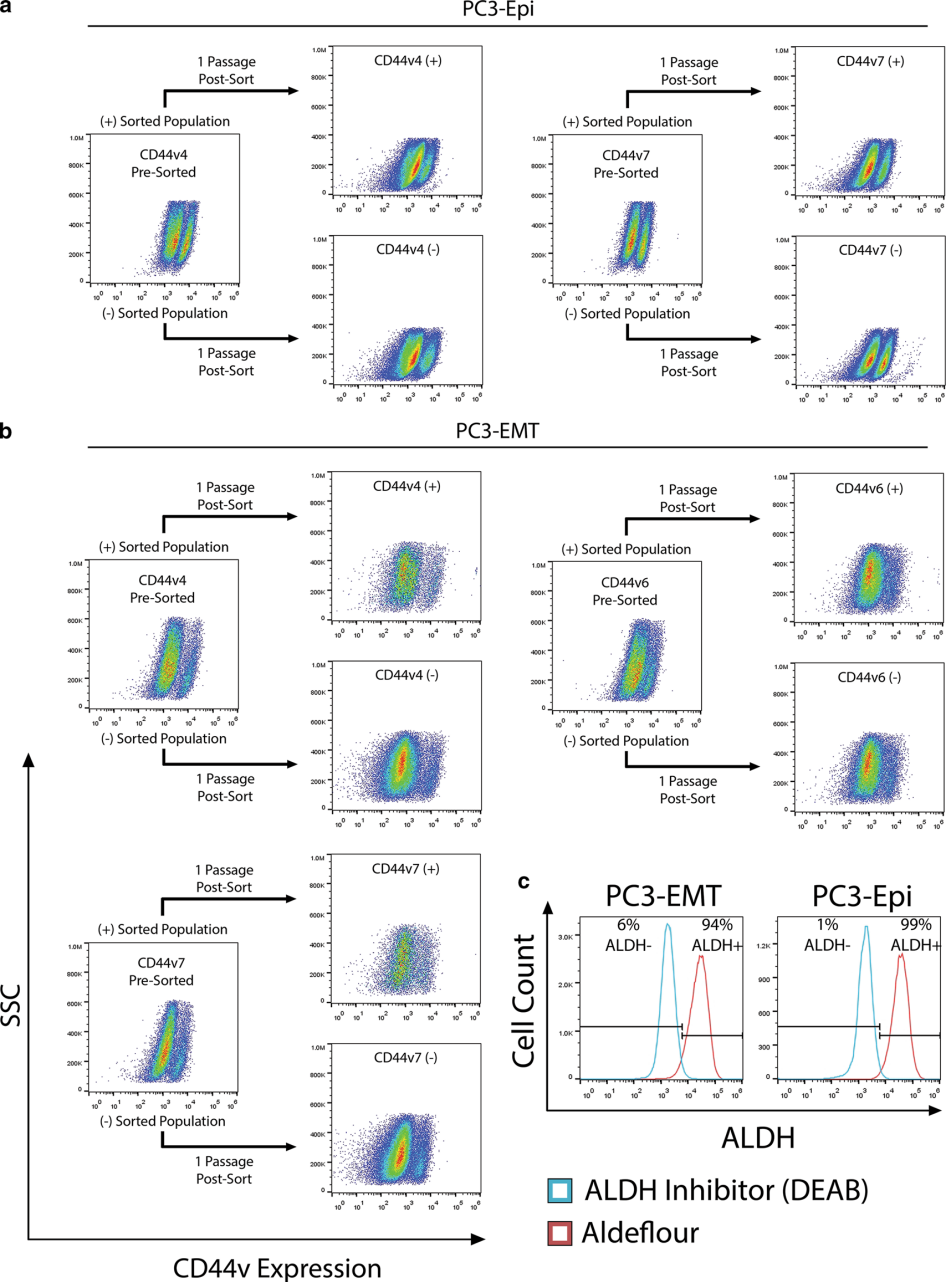
CD44 variant expression is plastic. **a** 10 % most positive and negative CD44v PC3-Epi populations were sorted by FACS and grown in culture for 23 days then reanalyzed for CD44v expression. Pre-sorted populations: 35 % CD44v4+ and 38 % CD44v7+. Post-sort populations: v4+ sorted population: 16 % CD44v4+, v4− sorted population: 19 % CD44v4+, v7+ sorted population: 25 % CD44v7+, v7− sorted population: 40 % CD44v7+. **b** 10 % most positive and negative CD44v PC3-EMT populations were sorted by FACS and grown in culture for 12 days and then reanalyzed for CD44v expression. Pre-sorted populations: 16 % CD44v4+, 18 % CD44v6+, and 16 % CD44v7+. Post-sorted populations: v4+ sorted population: 14 % CD44v4+, v4− sorted population: 9 % CD44v4+, v6+ sorted population: 11 % CD44v6+, v6− sorted population: 13 % CD44v6+, v7+sorted population: 18 % CD44v7+, v7− sorted population: 11 % CD44v7+. **c** ALDH activity in PC3-Epi and PC3-EMT determined by FACS using ALDEFLUOR kit

In order to determine whether expression of any of these CD44 variants represented a stable subpopulation, flow cytometry was used to isolate pure variant positive and negative populations. These six subpopulations (CD44v4∓, CD44v6∓ and CD44v7∓) all reverted back to a population containing similar ratios to the original PC3-Epi or PC3-EMT populations within a few weeks (Fig. 4). While the exact percentages are not displayed on the density plots (check legend), what should be noted is the similarity between positive and negatively sorted populations after the given time period. Also note that PC3-Epi CD44v6 is not shown because it does not have an applicable negative population. These data demonstrate that similar to E-cadherin (Fig. 1b), CD44 splice variant expression is stochastic. Some cells have higher splice variant expression than other cells, but splicing is not selectable by single cell cloning.

Much like CD44, the aldehyde dehydrogenase (ALDH) superfamily expression is also associated with CSCs and cancer progression. The 19 isozyme ALDH superfamily has been shown to perform a wide variety of biological functions, such as cell proliferation, differentiation and survival. Additionally, clinical studies of prostate cancer patients have demonstrated a negative correlation between ALDH expression and overall survival at 5 years [19]. Consequently, ALDH has emerged as an important marker used to isolate and investigate CSCs. ALDH activity was determined using the ALDEFLUOR kit (Stemcell Technologies). Flow cytometric analysis showed that 100 % of both PC3-Epi and PC3-EMT populations displayed high ALDH activity (Fig. 4c), while LNCaP had very low expression (Supplement 3). LNCaP was originally derived from an androgen sensitive lymph node metastasis [20]. Consequently, LNCaP is a much less aggressive line that the bone derived PC3 line.

## Discussion

In this work, we discovered that the expression of CD44 variant exon 6 (CD44v6) is greater in PC3-Epi than PC3-EMT. It was also very recently shown that CD44v6 expression is necessary for anchorage independent growth and that high expression is associated with a poor outcome in prostate cancer patients [21]. Additionally, other studies in lung, colon and breast indicate that stronger CD44v6 expression is a negative prognostic indicator [22–24]. In order to determine the relationship between EMT, CD44 isoforms and ALDH, we characterized PC3-Epi and PC3-EMT via qPCR, FACs and western blotting.

Using FACs, the CD44v6 variant was more highly enriched at the protein level in PC3-Epi than in PC3-EMT, but expression of CD44v4/7 was much less dramatic. This is somewhat surprising since the isoforms present in PC3-Epi/EMT (Fig. 3a) always have v4/v6/v7 coexpressed in the same isoforms (Fig. 3a). It is possible that the antibodies have a difficult time recognizing CD44v4 and CD44v7 as an epitope, possibly due to glycosylation or some other post-translational modifications that mask antibody binding sites [25, 26]. It is surprising that PC3-EMT elicits any CD44v6 expression since it is undetectable at the western and qPCR levels. In all likelihood, PC3-EMT will be shown to be null by flow cytometry when more specific antibodies are available.

Immunohistochemistry from various groups is inconclusive about the value of CD44v6 as a predictor for prostate cancer survival [27–29]. Given our FACs results, this is not surprising that immunohistochemistry only relies on immunoaffinity [30]. FACs lacks the second dimension that a gel sizing can add which reduces the specificity of flow cytometry. Our results demonstrate that many PC3-EMT have CD44v6; however, they clearly do not according to qPCR. Additionally, many PC3-Epi cells are negative for CD44v7 which is very unlikely given the sequence of the isoforms shown in the RNAseq data all have CD44v7 coexpressed with CD44v6.

Interestingly, it has been suggested that CD44v6 is important in drug resistance since knock down of CD44v6 prevented colony formation after exposure to various chemotherapeutics. Somewhat unexpectedly, however, knock down of CD44v6 also diminished the expression of mesenchymal markers, including vimentin, Snai1/2 and Twist. The authors suggest that this is due to decreased AKT activity with CD44v6 being a constituent of the WNT signaling pathway, a known inducer of EMT. This seems to argue with our data since all PC3-Epi expressed CD44v6, while only 50 % of PC3-EMT was CD44v6 positive by flow cytometry. However, it may be that CD44v6 is needed at low levels to stimulate WNT but at higher levels or when coexpressed with other variants it functions in alternative pathways, which do not induce EMT [21, 31].

In conclusion, once specific antibodies are made to either CD44v6 or one of the other variant exons, it is likely that it will be an excellent way to differentiate cells by flow cytometry. In the future, the establishment of antibodies needs to better take into account post-translational modifications, such as N/O-linked glycosylation, which are differentially utilized in mesenchymal vs. epithelial cells [32]. There has been much work that has shown that CD44v expression alone prevents EMT [9, 33], which means that CD44 splicing alone maybe just as good, if not better than cell adhesion proteins such as E-cadherin in predicting the differentiation status of cells. Unlike E-cadherin, which was only 40 % positive in a pure population, CD44v6 was 100 % positive in the PC3-Epi line, indicating that it may be a superior method to distinguish epithelial cells.

## Electronic supplementary material

Below is the link to the electronic supplementary material.

Parental PC3 prostate cancer cells are a mixture of cells. Expression of E-cadherin, vimentin and actin was assessed by western blotting (TIFF 10490 kb)

A) qPCR analysis of CD44v expression of PC3-EMT, PC3-Parental, and LNCaP relative to PC3-Epi. Results were normalized to β-actin; *p* values are represented as * p < 0.05. B) General CD44 expression in PC3-Parental and LNCaP determined by flow cytometric analysis. Unstained cells were used to measure cellular autoflorescence. C) Flow cytometric analysis of CD44v expression in PC3-Parental and LNCaP cells (TIFF 78604 kb)

Flow cytometric analysis of ALDH activity was determined using ALDEFLUOR in PC3-Parental and LNCaP cells using DEAB as a negative control (TIFF 13356 kb)

Antibodies used for western blotting (DOC 32 kb)

Antibodies used for flow cytometry (DOC 29 kb)
