# Supplementary figures and images for: Alternative CD44 splicing identifies epithelial prostate cancer cells from the mesenchymal counterparts
Source: Med Oncol. 2015 Apr 9;32(5):159. doi: 10.1007/s12032-015-0593-z (PMC4391735; doi:10.1007/s12032-015-0593-z)

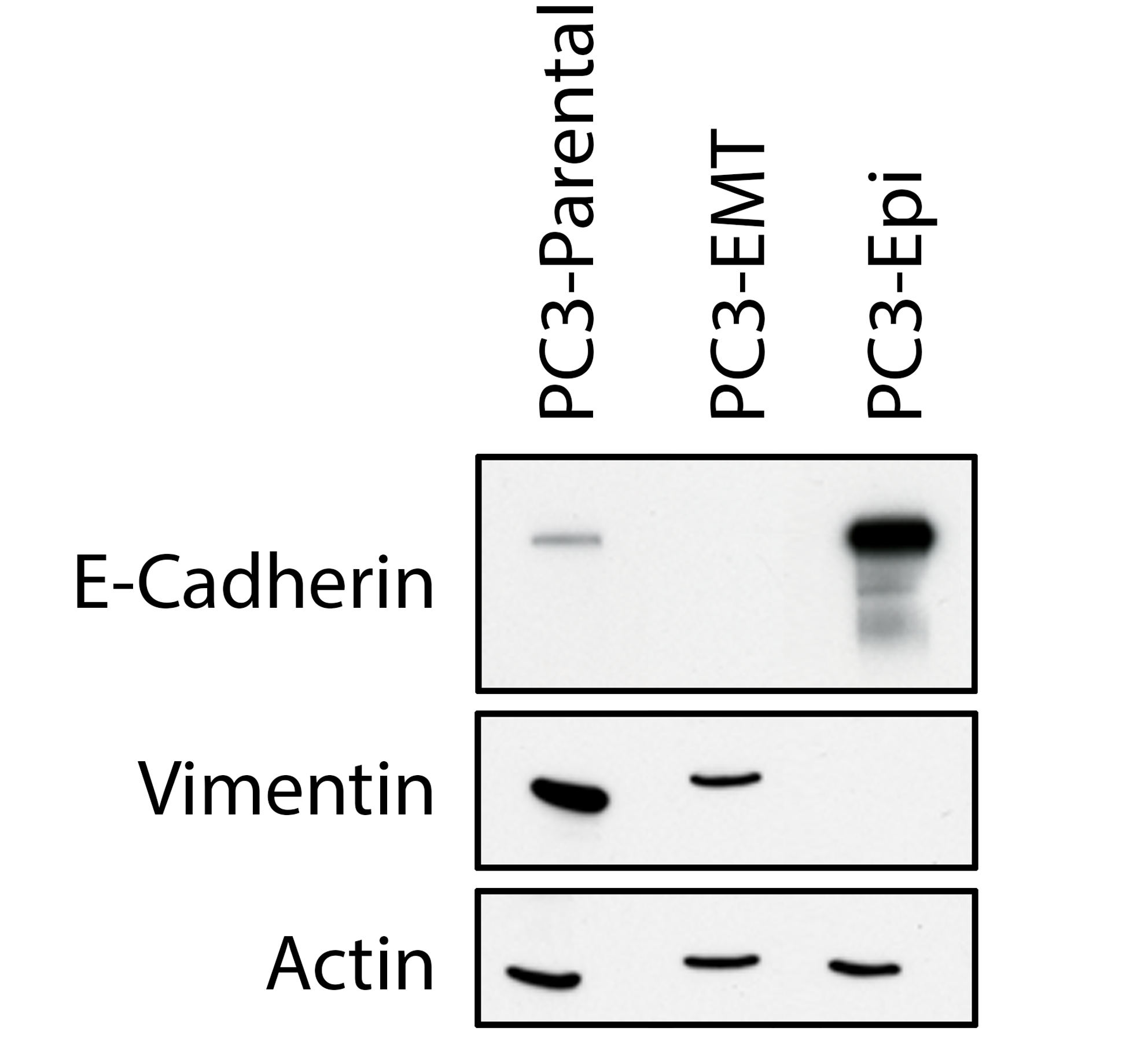

Supplement: Supplementary file 1 — Parental PC3 prostate cancer cells are a mixture of cells. Expression of E-cadherin, vimentin and actin was assessed by western blotting (TIFF 10490 kb) [file 12032_2015_593_MOESM1_ESM.tif]

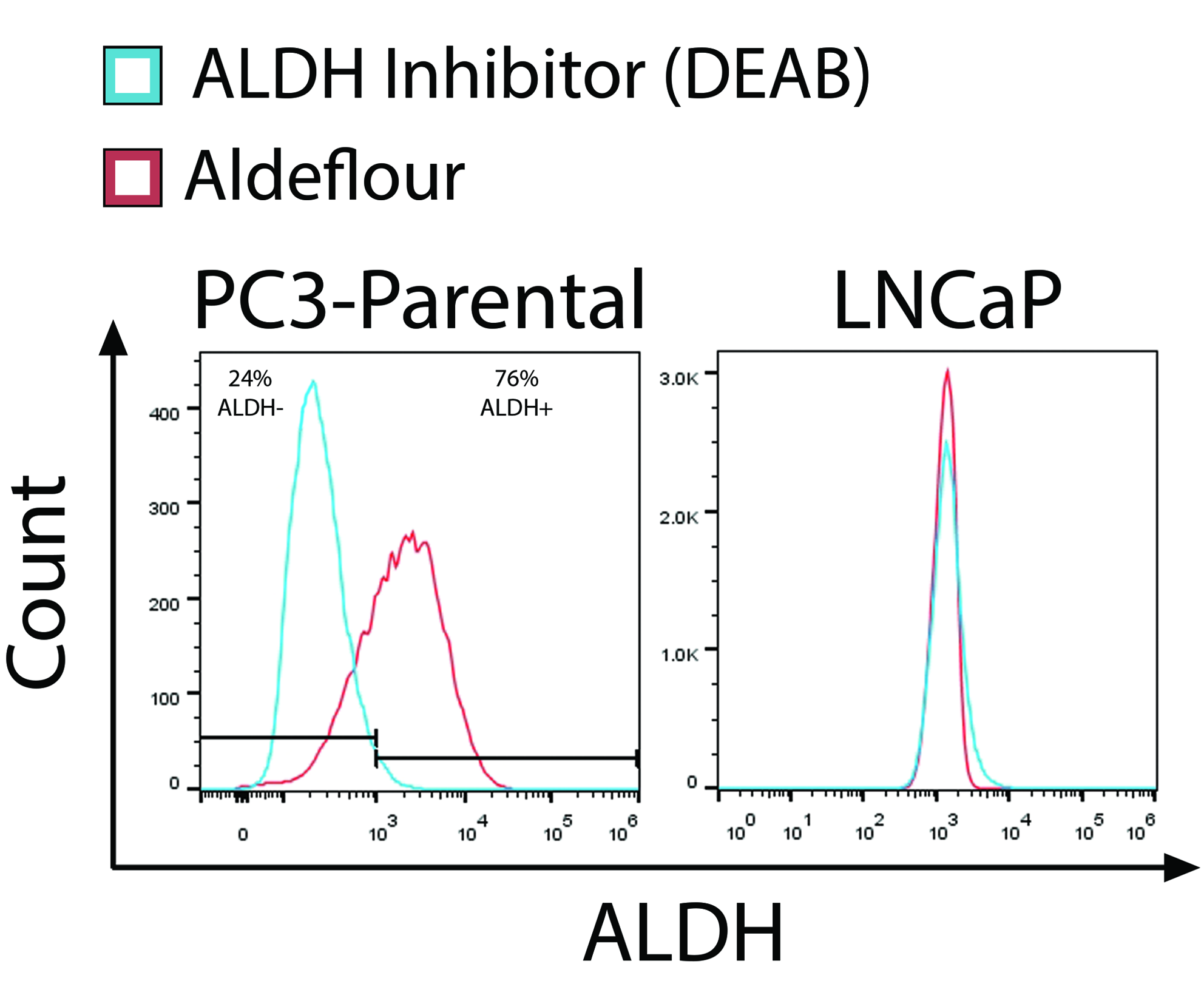

Supplement: Supplementary file 3 — Flow cytometric analysis of ALDH activity was determined using ALDEFLUOR in PC3-Parental and LNCaP cells using DEAB as a negative control (TIFF 13356 kb) [file 12032_2015_593_MOESM3_ESM.tif]
