# Supplementary material for: Alternative CD44 splicing identifies epithelial prostate cancer cells from the mesenchymal counterparts
Source: Med Oncol. 2015 Apr 9;32(5):159. doi: 10.1007/s12032-015-0593-z (PMC4391735; doi:10.1007/s12032-015-0593-z)
Supplement: Supplementary file 4 — Antibodies used for western blotting (DOC 32 kb) [file 12032_2015_593_MOESM4_ESM.doc]

| **Immunoblot Antibody Information** | | |
| --- | --- | --- |
| **Gene** | **Primary Antibody** | **Secondary Antibody** |
| ESRP1 | Anti-Rabbit Polyclonal (Sigma: HPA023719) | Goat anti-Rabbit IRDye 800LT (LI-COR: 926-32211) |
| KRT18 | Anti- Mouse Monoclonal (Santa Cruz Biotechnology: sc-6259) | Goat anti-Mouse IRDye 680LT (LI-COR: 926-68020) |
| KRT19 | Anti-Mouse Monoclonal (Sigma: C-7159) | Goat anti-Mouse IRDye 680LT (LI-COR: 926-68020) |
| CD44 | Anti-Mouse Monoclonal (Cell Signaling: 156-3C11) | Goat anti-Mouse IRDye 680LT (LI-COR: 926-68020) |
| E-Cadherin | Anti-Rabbit Monoclonal (Cell Signaling: 24E10) | Goat anti-Rabbit IRDye 800LT (LI-COR: 926-32211) |
| TCF8/ZEB1 | Anti-Rabbit Polyclonal (Sigma: HPA027524) | Goat anti-Rabbit IRDye 800LT (LI-COR: 926-32211) |
| Vimentin | Anti-Rabbit Monoclonal (Cell Signaling: D21H3) | Goat anti-Rabbit IRDye 800LT (LI-COR: 926-32211) |
| β-Actin | Anti-Mouse Monoclonal (Sigma: A5441) | Goat anti-Mouse IRDye 680LT (LI-COR: 926-68020) |
| α/β- Tubulin | Anti-Rabbit Polyclonal (Cell Signaling: 2148) | Goat anti-Rabbit IRDye 800LT (LI-COR: 926-32211) |
