# Supplementary material for: Alternative CD44 splicing identifies epithelial prostate cancer cells from the mesenchymal counterparts
Source: Med Oncol. 2015 Apr 9;32(5):159. doi: 10.1007/s12032-015-0593-z (PMC4391735; doi:10.1007/s12032-015-0593-z)
Supplement: Supplementary file 5 — Antibodies used for flow cytometry (DOC 29 kb) [file 12032_2015_593_MOESM5_ESM.doc]

| **Flow Cytometry Antibody Information** | |
| --- | --- |
| **Gene** | **Antibody** |
| CD44-v4 | Anti-Mouse Monoclonal (AbD Serotec: MCA1728F) |
| CD44-v6 | Anti- Mouse Monoclonal (AbD Serotec: MCA1730F) |
| CD44-v7 | Anti-Mouse Monoclonal (AbD Serotec: MCA1730F) |
| E-Cadherin | Anti-Rabbit Monoclonal (Cell Signaling: 24E10) |
| N-Cadherin | Anti-human CD325 (N-Cadherin) (BioLegend: 350806) |
